# Application and interpretation of core elements of the 2015 NMOSD diagnostic criteria in routine clinical practice

**DOI:** 10.3389/fimmu.2024.1515481

**Published:** 2024-12-13

**Authors:** Edgar Carnero Contentti, Juan I. Rojas, Ricardo Alonso, Michael R. Yeaman, Brian G. Weinshenker

**Affiliations:** ^1^ Neuroimmunology Unit, Department of Neurosciences, Hospital Aleman, Buenos Aires, Argentina; ^2^ Centro de Enfermedades Neuroinmunológicas de Rosario (CENRos), Neuroimmunology Clinic, Instituto de Neurologia Cognitiva (INECO) Neurociencias Oroño, Rosario, Argentina; ^3^ Department of Neurology, Hospital Universitario Centro de Educación Médica e Investigaciones Clínicas (CEMIC), Buenos Aires, Argentina; ^4^ Department of Neurology, Hospital Ramos Mejia, Buenos Aires, Argentina; ^5^ Department of Medicine, Divisions of Molecular Medicine and Infectious Diseases, David Geffen School of Medicine, University of California, Los Angeles, Los Angeles, CA, United States; ^6^ Department of Medicine Lundquist Institute for Biomedical Innovation at Harbor-University of California Los Angeles Medical Center, Los Angeles, CA, United States; ^7^ Department of Neurology, University of Virginia, Charlottesville, VA, United States

**Keywords:** neuromyelitis optica spectrum disorder, diagnosis, misdiagnosis, criteria, MRI

## Abstract

**Background:**

We evaluated comprehension and application of the 2015 neuromyelitis optica spectrum disorder (NMOSD) criteria core elements by neurologists in Latin America (LATAM) who routinely diagnose and care for NMOSD patients by (i) identifying typical/suggestive NMOSD syndromes, (ii) detecting typical MRI NMOSD lesions and meeting MRI dissemination in space (DIS) criteria, and (iii) evaluating historical symptoms suggestive of NMOSD.

**Methods:**

We conducted an anonymous, voluntary, self-administered web- and case-based survey cross-sectional study from October 2023 to January 2024 of neurologists identified through the LACTRIMS database. Questions were presented first through iterative clinical cases or imaging, followed by questions directly evaluating comprehension of definitions. “Correct” responses were based on the 2015 criteria and adjudicated by the consensus of the experts leading the project.

**Results:**

A total of 106 neurologists (60.3% female; mean age: 46.6 ± 12.5 years) were included. Between 10.4% and 49.1% of neurologists inaccurately identified clinical or paraclinical aspects for DIS and 32.1% accurately identified the three non-cardinal (brainstem, diencephalic, and cerebral) syndromes for seronegative patients. Between 35.8% and 64.1% of neurologists identified the “optimal timing” of AQP4-IgG testing (e.g., during an attack or before receiving immunosuppressant treatments, among others); 56.6% considered live cell-based assay as the gold standard method for serological testing. Most neurologists accurately identified typical NMOSD MRI lesions, but periventricular, juxtacortical/cortical, fluffy infratentorial, corticospinal tract, and hypothalamic lesions were frequently misidentified.

**Conclusion:**

Clinical scenarios were identified where the 2015 NMOSD criteria were susceptible to misinterpretation and misapplication by expert neurologists in LATAM. Implementing collaborative educational initiatives could improve NMOSD diagnosis and raise patient care standards.

## Introduction

Neuromyelitis optica spectrum disorder (NMOSD) is a rare but debilitating inflammatory and immune-mediated disease of the central nervous system (CNS) linked to the presence of disease-specific, pathogenic aquaporin 4-antibodies (AQP4-IgG) in the majority of patients (80%) (1, 2). Differentiating NMOSD from its mimics is critical to reduce misdiagnosis, particularly in patients with negative or unknown AQP4-IgG testing (3). Multiple sclerosis (MS) and other immune-mediated conditions such as myelin oligodendrocyte glycoprotein antibody (MOG-IgG)-associated disease (MOGAD) are important differential diagnoses in clinical practice (4–6). Other rare or different conditions including infectious, metabolic, and vascular diseases can also present with similar symptoms at disease onset or during follow-up, such as transverse myelitis (TM), optic neuritis (ON), and attacks on the brainstem and/or the brain (6, 7). There may also be overlapping paraclinical and neuroradiological features observed, especially with MOGAD (7, 8). However, the 2015 NMOSD criteria did not explicitly address the differentiation of MOGAD from AQP4-IgG-positive NMOSD or MS. Overall, the diagnosis of NMOSD depends on successive clinical assessments (1, 2). An early diagnosis of NMOSD is crucial to improve long-term patient outcomes. There has been a concerted effort over the past 25 years to update and improve diagnostic criteria to facilitate earlier and more precise diagnosis (1, 2). Certain magnetic resonance imaging (MRI) lesions have been reported to be typical or suggestive of NMOSD, such as those occurring in the dorsal medulla and hypothalamus (1, 2). Among others, 37% of NMOSD patients may present with lesions that are characteristic of MS, fulfilling the 2017 criteria for dissemination in space (DIS) (9). Making a rapid and accurate diagnosis of NMOSD is crucial, as delays in acute or long-term therapeutic strategies may result in worsened prognosis and disability in NMOSD (10). AQP4-IgG and MOG-IgG tests are important for diagnosing antibody-mediated disorders, but they are not easily accessible in all countries (11). Thus, results are often delayed, limiting the contribution of this test to the differential diagnosis process (12). Unlike NMOSD, no specific biomarker for MS diagnosis has been identified in clinical practice (4). In this context, patients without MS (e.g., NMOSD) can be misdiagnosed with MS, despite following validated international diagnostic criteria (13). The NMOSD diagnostic criteria have evolved over time, including better characterization of serum tests, neuroimaging lesions, and well-defined clinical core characteristics (1, 2). NMOSD diagnosis is mainly based on accurately interpreting symptoms, disease history, neurologic examination, laboratory tests, and neuroradiological information (2). In addition, the 2015 NMOSD diagnostic criteria emphasized on the need to rule out any “other better explanation or alternative diagnoses” for the clinical scenario before making a definitive diagnosis of NMOSD (2). Application of these criteria in the Latin America (LATAM) population has resulted in a 62.5% increase in incidence of NMOSD diagnosis as compared to the 2006 NMO criteria, with a shorter median time to diagnosis (14). However, a recently published study found that 12% (56 out of 469 with an initial diagnosis other than NMOSD) of LATAM patients who had been referred for care with another previously established diagnosis had been misdiagnosed (i.e., the incorrect diagnosis of patients who truly have NMOSD) (13). This relatively low rate of misdiagnosis is a considerable improvement from historical rates of misdiagnosis, which were 50% or greater (15–19). Nonetheless, in that large LATAM cohort, misinterpretation and misapplication of core elements (clinical and neuroradiological aspects) of the 2015 NMOSD diagnostic criteria led to misdiagnosis of NMOSD (13). Consequently, in many cases, NMOSD misdiagnosis was associated with inappropriate treatment, leading to suboptimal patient benefit that potentially worsened disability and enabled relapses, along with promoting the unnecessary use of health resources in the region. However, there are no studies evaluating knowledge gaps for application of core elements of the 2015 NMOSD criteria by attending clinicians in LATAM countries. In this study, we evaluated comprehension and application of the 2015 NMOSD criteria core elements by neurologists in LATAM who routinely diagnose and care for NMOSD patients.

## Methods

A cross-sectional study was conducted from October 2023 to January 2024. An anonymous, voluntary, self-administered web- and case-based survey was conducted by coordinating investigators of the study (the survey is displayed in the **Supplementary Materials**).

The survey was available online for 4 months and was developed in both Spanish and English. Before distribution via email, the pilot English survey was reviewed by an international expert in NMOSD (B.W.) to ensure that the items accurately addressed the research questions. Additionally, five neurologists from LATAM tested the survey, who were not involved in designing the survey and did not participate in the study. Participants were identified through the Latin American Committee for Treatment and Research in MS (LACTRIMS) database, including several countries’ working groups. Brazilian neurologists received the survey in English, and the rest of the participants received the survey in Spanish. Responses were securely collected online, via Google forms. Reminder emails were sent every 2 weeks via LACTRIMS mailing.

Participants were instructed not to review the NMOSD criteria while performing the survey. “Correct or accuracy” responses were based on the 2015 diagnostic criteria and adjudication by the consensus of the experts leading the project. To minimize learning effect, questions designed to evaluate criteria interpretation and application were presented first through iterative hypothetical and fictional clinical case examples for the survey using multiple choice or imaging, followed by questions directly evaluating comprehension of definitions. Except for questions with ordinal responses, potential responses were randomly ordered for each participant.

The purpose of this survey was to assess the understanding, interpretation, and application of the key elements of the 2015 NMOSD diagnostic criteria by (i) identifying typical or suggestive NMOSD syndromes, (ii) detecting typical MRI NMOSD lesions and meeting MRI DIS criteria, and (iii) evaluating historical symptoms suggestive of NMOSD.

This study was approved by the Independent Ethics Committee of the “Hospital Alemán de Buenos Aires”. All participants signed an electronic informed consent form before data collection.

For this study, we followed the Guidelines from Strengthening the Reporting of Observational Studies in Epidemiology (STROBE), as shown in the **Supplementary Materials**.

### Statistical analysis

The data analysis was carried out using the STATA program. Descriptive statistics were presented as mean ± standard deviation (SD), median value, and percentages to evaluate the diversity of the cohort study. Because of the exploratory nature of the study, no sample size calculations were performed.

## Results

We surveyed 123 participants, including 3 ophthalmologists. Fourteen surveys were incomplete in more than 60% of their content. A total of 106 responses from neurologists were included and analyzed. Incomplete surveys and those from ophthalmologists were excluded (*N* = 17). The survey was sent to approximately 450 LATAM neurologists; however, we were unable to calculate a response rate due to both design and diffusion of the survey (total number of recipients of the study survey email invitation was unknown).

### Neurologist demographic profile

As indicated in **Table 1**, 60.3% of participants were women, with a mean age of 46.6 ± 12.5 years. Respondents were from Argentina (28.3%), Brazil (19.2%), Colombia (12.2%), and 11 additional countries (40.3%) (**Supplementary Table 1**). Respondents were 13.7 ± 11 years post-graduation, of which 8 ± 3 years were dedicated to NMOSD healthcare after completing their training. On average, neurologists diagnose 7.1 ± 18.3 NMOSD new cases per year (ranging from 1 to 50), with most of them (37.2%) practicing in an academic medical center.

**Table 1:** Baseline characteristics.

	*N* = 106
**Gender, *n* (%)** Female	64 (60.3)
**Mean age (years) ± SD (range), years**	46.6 ± 12.5 (30–78)
**Countries, *n* (%)** Argentina Brazil Colombia Others*	30 (28.3) 21 (19.2) 13 (12.2) 42 (40.3)
**Years post-graduation, mean ± SD (range)**	13.7 ± 11 (3–45)
**For how many years following completion of your training have you cared for patients with NMOSD?, mean ± SD (range), years**	8 ± 3 (1–24)
**Practice type, *n* (%)** Academic medical center Individual private practice Group private practice Other	45 (37.2) 34 (28.1) 32 (26.4) 10 (8.3)
**Percentage of your professional time involved in clinical activities, *n* (%)** 25% or less 26%–50% 51%–75% Greater than 75%	17 (16.1) 20 (18.8) 59 (55.6) 10 (9.5)
**Percentage of the patients for which you provide ongoing care have:** **NMOSD or CNS inflammatory disease other than MS, *n* (%)** 1%–5% 6%–15% 16%–30% Greater than 30% **MS, *n* (%)** 25% or less 26%–50% 51%–75% Greater than 75%	36 (33.9) 35 (33.1) 21 (19.8) 14 (14.2) 37 (34.9) 34 (32.1) 18 (16.9) 17 (16.1)
**Estimate number of new NMOSD diagnoses per year, mean ± SD (range)**	7.1 ± 18.3 (1–50)

*All included countries are shown in the **Supplementary Materials**.

Correct answers are shown in bold.

### Access to educational meeting or training from LATAM neurologists

As shown in **Supplementary Table 2**, 64 (60.3%) participants underwent training focused on neuroimmunology, of whom 46.9% received training in referral centers from LATAM. The majority of LATAM neurologists expressed a desire to access international educational meetings/training (78.3%), but a significant portion of them (70.8%) are unable to do so for financial reasons. Most neurologists (91.5%) find the 2015 NMOSD criteria easy to comprehend and apply in clinical practice, with most of them (87.7%) carefully reviewing the manuscript for the 2015 IPND NMOSD criteria more than twice, at least 1 month apart.

### Comprehension and application of the 2015 NMOSD criteria

The initial case vignette (Case #1) featured a patient with a history of neuromyelitis (ON+TM). She was currently experiencing myelopathy associated with LETM on spinal MRI, illustrating a typical case of NMOSD (**Table 2**). Brain MRI did not reveal any lesions. Most (98.1%) neurologists accurately recognized that this patient exhibited findings commonly observed in NMOSD patients seropositive for AQP4-IgG. Additionally, 89.6% of neurologists accurately identified the concept of DIS in a patient with a history of confirmed ON and TM with poor recovery, and 95.5% correctly identified the application of the 2015 NMOSD criteria for seronegative patients. Nonetheless, participants were asked if a history of ON would not have been present, but VEP was prolonged (despite the absence of clinical symptoms in that eye), which would have satisfied DIT in this patient, and 49.1% incorrectly responded that VEP prolongation in the absence of objective evidence of an attack of ON would satisfy the criteria for diagnosis.

**Table 2:** Case #1.

A 39-year-old woman with one prior optic neuritis in the right eye (confirmed by an ophthalmologist) and one prior myelitis event with moderate residual weakness of the right leg. Currently, she consults for severe weakness in both legs associated with sphincter disorders that was confirmed by the neurologist during examination. No other sign and symptoms. **Brain and spinal cord MRI:** Brain MRI does not reveal any new T2 or enlarging gadolinium enhancing lesions. Cervical and thoracic spinal cord MRI show a new thoracic T2 lesion extending from T1 to T8 with gadolinium enhancement from T3 to T5.	
**What is your opinion?, *n* (%)** These findings are commonly seen in MS patients **These findings are commonly seen in patients with NMOSD seropositive for AQP4-Ab** These clinical manifestations are commonly seen in patients with NMOSD, but not the MRI findings These clinical manifestations are not commonly seen in patients with NMOSD, but spinal MRI lesion is typically seen in NMOSD.	0 **104 (98.1)** 0 2 (1.9)
**Does this patient’s presentation fulfill dissemination in space criteria for a diagnosis of NMOSD?, *n* (%)** **Yes** No Not reported	**95 (89.6)** 2 (1.8) 9 (8.6)
**If a history of optic neuritis would not have been present, but visual evoked potential of the right eye was prolonged despite absence of clinical symptoms in that eye, would criteria for NMOSD diagnosis been satisfied?, *n* (%)** **No** Yes Do not know Not reported	**54 (50.9)** 44 (41.5) 5 (4.9) 3 (2.7)
**Based on the data above, does this patient meet diagnostic criteria for NMOSD if seronegative for AQP4-IgG?, *n* (%)** No, this patient does not have a core clinical characteristic of NMOSD. No, although this patient presents with a core clinical characteristic for NMOSD, the patient is AQP4-IgG seronegative. **Yes, this patient presents with a core clinical characteristic for NMOSD, and history of other core clinical characteristics and has the necessary supportive MRI finding of an acute longitudinally extensive spinal cord lesion thus meeting dissemination in space criteria assuming that no better explanation exists even with AQP4-Ab negative/unknown.** No, this patient presents with a core clinical characteristic for NMOSD, but dissemination in space and dissemination in time criteria are not fulfilled, and therefore, the patient has no NMOSD.	2 (1.8) 2 (1.8) **101 (95.5)** 1 (0.9)

Correct answers are shown in bold.

As shown in **Table 3**, the majority (86.8%) of LATAM neurologists accurately noted that the radiological presentation was uncommon and atypical [Case #2; history of ON with current short transverse myelitis (STM) on MRI]. However, they accurately mentioned that this does not exclude the diagnosis of NMOSD. In the other case, most participants accurately pointed out that the clinical presentation was typical for NMOSD [Case #3, patients presenting with area postrema syndrome (APS), which is a core clinical characteristic]. Neurologists accurately identified that both patients should be tested for AQP4-IgG to reach NMOSD diagnosis.

**Table 3:** Case #2 and Case #3.

A 33-year-old woman with one prior optic neuritis in the right eye with poor recovery (confirmed by an ophthalmologist). Currently, she consults for moderate weakness in both legs (asymmetric with left predominance) associated with sphincter disorders that was confirmed by the neurologist during examination. No other sign and symptoms. **Brain MRI** shows a short canalicular lesion in the right ON in T2 without gadolinium enhancing and brain was normal. **Cervical and thoracic spinal cord MRI** show a thoracic T2 lesion from T3 to T4 (central in the axial plane) with gadolinium enhancement at T3.	
**Should the patient be tested for AQP4-IgG?, *n* (%)** No, this patient does not have a core clinical characteristic of NMOSD. **Yes, this patient had a history of a core clinical characteristic of NMOSD and she now has clinically and radiologically a short-transverse myelitis, which does not rule out NMOSD.** This patient has a short-transverse myelitis on spinal MRI, which rules out NMOSD. No, this patient had a history of ON typical of MS and she now has clinically and radiologically a short-transverse myelitis, which rules out NMOSD. Not reported	7 (6.6) **92 (86.8)** 3 (2.8) 3 (2.8) 1 (0.9)
A 26-year-old woman with no relevant medical history of prior diseases. She developed intractable episodic nausea and vomiting that evolved over 3 days. She was evaluated by a gastroenterologist, but no improvement after symptomatic therapy was observed. The patient was treated with intravenous methylprednisolone with complete recovery. **Brain MRI** shows bilateral lesion involving the dorsal medulla. **Cervical and thoracic spinal cord MRI** was normal.	
**Should the patient be tested for AQP4-IgG?, *n* (%)** **Yes, this patient has a core clinical characteristic of NMOSD associated with area postrema lesions on MRI.** No, this patient does not have a core clinical characteristic of NMOSD, and false positive serological testing is common in this setting. Yes, although this patient does not fulfill criteria for an area postrema syndrome. No, this patient has a typical syndrome of MS.	**101 (95.2)** 1 (0.9) 3 (2.8) 1 (0.9)

Correct answers to each question are shown in bold.

### Identification of typical clinical syndromes and MRI features for NMOSD

Most neurologists inaccurately identified all three non-cardinal/common core clinical characteristics (brainstem + diencephalic + cerebral syndromes) for NMOSD to accurately evaluate the main clinical manifestation supporting a diagnosis of NMOSD in AQP4-IgG-seronegative cases. Over 77% of LATAM neurologists correctly identified typical clinical presentations of NMOSD or highly suggestive for NMOSD (5 out of 14), while atypical presentations were chosen by at least 33% (**Table 4**). Many participants missed typical/suggestive NMOSD MRI lesions, which are also additional MRI requirements for NMOSD with negative AQP4-IgG and NMOSD with unknown AQP4-IgG status, except for ON, myelitis, and APS lesions. Simultaneously, we assessed the same concepts regarding AQP4-IgG, revealing that between 35.8% and 64.1% of neurologists correctly identified the optimal moment to request AQP4-IgG (e.g., during an attack or before receiving immunosuppressant treatments, among others) and 56.6% considered the live cell-based assay as the gold standard method to improve the sensitivity and to avoid false-negative cases. However, tissue-based indirect immunofluorescence (IIF) (4.7%) and enzyme-linked immunosorbent assay (ELISA) (4.7%) were incorrectly selected as the gold standard method. Regarding the clinical practice aspects of treating neurologists before making a new diagnosis of NMOSD, most participants request for general lab tests and spinal cord MRI, while approximately half undergo CSF testing and orbital MRI (**Supplementary Table 3**). The proportion of responses for AQP4-IgG, typical clinical syndromes, and MRI features for NMOSD are summarized in **Table 4**.

**Table 4:** Correct identification of typical clinical syndromes and MRI features for NMOSD.

	N (%)
Although considered a core clinical characteristic for NMOSD, which manifestation **is not a cardinal syndrome** and **would be insufficient to support a diagnosis of NMOSD in an AQP4-IgG-seronegative** patient for NMOSD? (Please select all that apply) Optic neuritis (ON) Transverse myelitis (TM) **Brainstem syndrome (BSS)** Area postrema syndrome (APS) **Diencephalic syndrome (DS)** **Symptomatic cerebral syndrome (SCS)** **Correct (Brainstem + Diencephalic + Cerebral syndromes)** Incomplete (2 out of 3 syndromes) without including ON/TM/APS Incomplete (1 out of 3 syndromes) without including ON/TM/APS Incomplete (2 out of 3 syndromes) including ON/TM/APS Incomplete (1 out of 3 syndromes) including ON/TM/APS	8 (7.5) 9 (8.4) 52 (49.1) 10 (9.4) 57 (53.8) 89 (83.9) **34 (32.1)** 26 (24.5) 29 (27.3) 6 (5.6) 3 (2.8)
**Which of the following are considered typical clinical presentations of NMOSD or highly suggestive for NMOSD?. Correct answers in bold.** **Acute unilateral optic neuritis with a poor visual recovery** **Complete transverse myelopathy with bilateral motor and sensory involvement** Double vision due to an internuclear ophthalmoplegia (in a young adult <40 years old) Headache or meningism Facial sensory loss or trigeminal neuralgia (in a young adult <40 years of age) Partial myelopathy Complete gaze palsy or fluctuating ophthalmoparesis Isolated fatigue or asthenia **Bilateral optic neuritis or unilateral optic neuritis with a poor visual recovery** Subacute cognitive decline **Intractable nausea, vomiting, or hiccoughs** Lhermitte’s symptom Urge incontinence or erectile dysfunction **Hypersomnolence or narcolepsy-like syndrome**	**82 (77.3)** **83 (78.3)** 8 (7.5) 0 3 (2.8) 18 (16.9) 6 (5.6) 4 (3.8) **100 (94.3)** 3 (2.8) **100 (94.3)** 20 (18.8) 35 (33.1) **85 (80.1)**
Which of the following regions may be used **as additional MRI requirements for NMOSD without AQP4-Ab and NMOSD with unknown AQP4-Ab**? (Check all that apply) **Long optic nerve lesion(s)** **Extensive periependymal brain lesions** Periventricular **Lesions involving the hypothalamus, thalamus** **Large, confluent subcortical or deep white matter lesion(s)** Cortical lesions **Lesions involving the dorsal medulla** Infratentorial lesions (i.e., middle cerebellar peduncles) Fluffy infratentorial lesions Subcortical **Long, diffuse, heterogeneous, or edematous corpus callosum lesions** Juxtacortical **Spinal cord (i.e., lesions extending over 3 or more complete vertebral segments)** **Periependymal lesions overlying the fourth ventricle** Periventricular lesions extending perpendicularly from ventricles into brain white matter **Long lesions in corticospinal tract pathway**	**97 (91.5)** **53 (50)** 4 (3.7) **58 (54.7)** **25 (23.6)** 2 (1.8) **69 (65.1)** 15 (14.1) 17 (16.1) 2 (1.8) **20 (18.8)** 0 **104 (98.1)** **72 (67.9)** 3 (2.8) **47 (44.3)**
**To fulfill NMOSD diagnostic criteria, seronegative patients must experience 2 or more different core clinical characteristics (i.e., dissemination in space, affecting different neuroanatomic regions) and other supportive MRI characteristics must also be present** False **True** Not reported	9 (8.4) **95 (89.6)** 2 (14)
Acute myelitis with extension into the brainstem can establish DIS (two different neuroanatomic regions)* **False** True Not reported	**10 (47.6)** 10 (47.6) 1 (4.8)
In clinical practice, diagnosis of NMOSD is not possible if OC bands are found with a pattern II or III (e.g., in a patient with severe ON and poor recovery + positivity for OCB)* **False** True Not reported	**18 (85.7)** 2 (9.5) 1 (4.8)
**Which of the following serologic tests would you perform if a phenotype of NMOSD is observed?** AQP4-Ab only in serum MOG-Ab only in serum **AQP4-Ab and MOG-Ab in serum (at the same time if possible)** AQP4-Ab only in CSF MOG-Ab only in CSF AQP4-Ab and MOG-Ab in serum and CSF (at the same time) AQP4-Ab and MOG-Ab in CSF (at the same time)	47 (44.3) 33 (31.1) **68 (64.1)** 1 (0.8) 0 16 (15.1) 1 (0.9)
When (optimal timing) would you request AQP4-Ab during the NMOSD diagnosis process (before making a new diagnosis of NMOSD)? (Check all that apply) *responses will be randomly ordered per respondent **During an attack** After receiving immunosuppressant treatments In remission phase **90 days after completion of treatment if PLEX or IVMP was received** **Before receiving immunosuppressant treatments** **After 3–6 months if initial AQP4-Ab were negative, but high suspicion of NMOSD exists.** Before 30 days if PLEX or IVMP was received	**66 (62.2)** 1 (0.9) 9 (8.4) **38 (35.8)** **68 (64.1)** **63 (59.4)** 7 (6.6)
What is the optimal method for testing AQP4-IgG to maximize sensitivity and specificity? Tissue-based IIF **Live cell-based assay** ELISA Fixed cell-based assay Flow cytometry Not reported	5 (4.7) **60 (56.6)** 5 (4.7) 6 (4.9) 5 (5.6) 25 (23.5)

*These questions were only answered by neurologists from Brazil (*N* = 21; survey in English). Missing data from the Spanish survey were found only for these questions.

Correct answers are shown in bold.

### Identification and comprehension of MRI features for NMOSD

**Figure 1:**
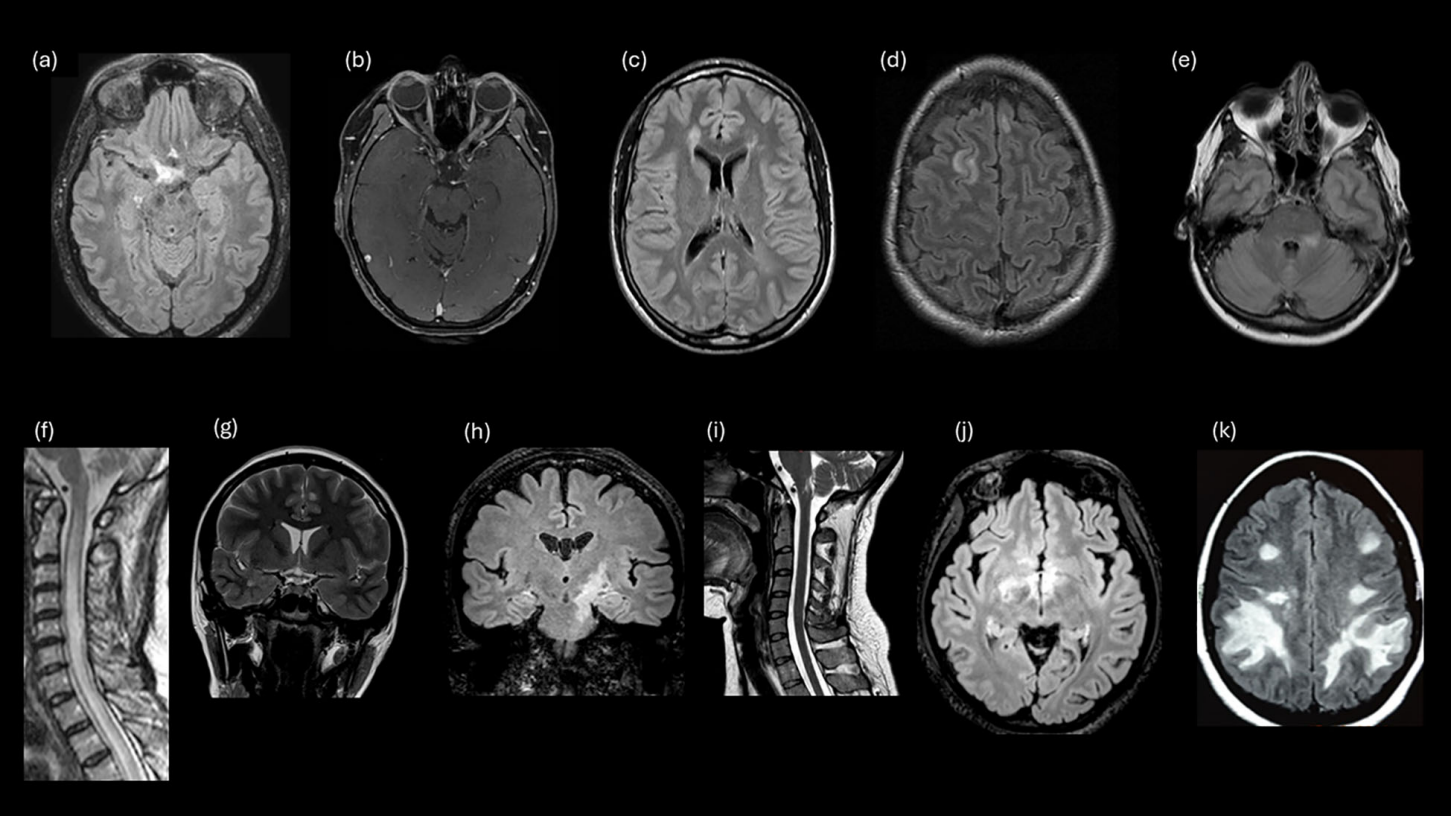
Examples of study survey images that evaluated participant knowledge for MRI lesions. **(A)** Lesion involving optic chiasm and posterior ON. **(B)** LEON (longitudinally extensive left optic neuritis). **(C)** Periventricular (MS). **(D)** Juxtacortical (MS). **(E)** Fluffy lesion and poorly demarcated lesion (MOGAD). **(F)** LETM. **(G)** Cortical lesion lob temporal (MS). **(H)** Corticospinal tract lesion. **(I)** Lesion involving the area postrema. **(J)** Hypothalamic lesion. **(K)** Large, confluent, bilateral subcortical or deep white matter lesions.

Neurologists were asked to identify typical lesions for NMOSD and type of lesions on 11 T2/FLAIR and T1 with and without contrast conventional MRI based on real cases. As shown in **Table 5** and **Figure 1**, most neurologists accurately identified typical NMOSD MRI lesions, but periventricular, juxtacortical/cortical, fluffy infratentorial, corticospinal tract, and hypothalamic lesions were frequently misidentified and misclassified as typical or atypical for NMOSD (**Table 5**). Based on the lesion type and its classification as typical or atypical, we observed adequate recognition of lesions associated with the three cardinal syndromes (ON, TM, and APS). However, while all neurologists correctly identified LETM, only 41.5% classified it as typical of NMOSD. Similarly, 63.2% classified the lesion in the APS as typical of NMOSD.

**Table 5:** Correct identification of typical or atypical MRI features for NMOSD.

Image *N*, %	a	b	c	d	e	f	g	h	i	j	k
**Typical lesion for NMOSD? (yes or no)** **Correct**	97 (91.5)	98 (92.4)	24 (22.6)	100 (94.3)	98 (92.4)*	44 (41.5)	14 (13.2)	25 (23.5)	67 (63.2)	91 (85.8)	82 (77.3)
**Type of MRI lesion?** **Correct**	88 (83.1)	103 (97.1)	70 (66.1)	77 (72.6)	2 (1.8)*	106 (100)	17 (16.1)	53 (50)	79 (74.5)	62 (58.4)	97 (91.5)

We requested the neurologists to identify the images (MRI) and then ascertain if the lesion is characteristic of NMOSD. A list with various options was given for the participants to select the accurate type of lesion. We displayed numbers and percentages of correct responses for each image. The images can be observed in **Figure 1**.

*A total of 92 responders chose infratentorial lesions (middle cerebellar peduncles).

Correct answers are shown in bold.

## Discussion

Over the past decade, there has been a profound improvement in clinical diagnosis and therapy for patients with NMOSD. However, room for improvement remains, particularly with respect to rapid and accurate diagnosis. In this study, we evaluated comprehension and application of the 2015 NMOSD criteria core elements by neurologists in LATAM who routinely diagnose and care for NMOSD patients. The findings revealed clinical scenarios with propensity for misunderstanding and/or misapplication of these 2015 NMOSD criteria. Areas of particular vulnerability to misdiagnosis of NMOSD syndromes were recognition of prototypical MRI NMOSD lesions, meeting MRI DIS criteria, and evaluating historical symptoms suggestive of NMOSD. In addition, most neurologists were less accurate in identifying all three non-cardinal core clinical characteristics for NMOSD (i.e., brainstem, diencephalic, and cerebral syndromes). Supporting the view that interpretation of imaging is a particularly challenging aspect of diagnosis, many of the study neurologists did not accurately identify some typical/suggestive NMOSD MRI lesions in AQP4-IgG-seronegative and unknown status, except for ON, myelitis, or APS lesions (2). For example, periventricular, juxtacortical/cortical, fluffy infratentorial, corticospinal tract, and hypothalamic lesions were frequently misidentified and misclassified in MRI. Recognizing clinical and radiologic requirements is important, particularly in AQP4-IgG-seronegative or in those with unknown results, because the 2015 diagnostic criteria require at least two core clinical characteristics supported by specific MRI findings to establish a diagnosis (2–6). One of these characteristics must be a cardinal manifestation such as ON, TM (with LETM MRI lesion), or APS (with associated medullary MRI lesion). Therefore, the 2015 criteria can be satisfied by a single clinical event even in an AQP4-IgG-seronegative patient with the requisite areas of neuroanatomic involvement (2).

Integration of clinical, serological, and radiological findings is essential in diagnosing NMOSD and related diseases. Alternative diagnoses and red flags that require extensive investigation should be considered before a conclusive diagnosis of NMOSD (6, 15) to avoid misdiagnoses (12). The primary consideration for differential diagnosis in NMOSD patients seronegative for both AQP4-IgG and MOG-IgG measured by CBA, termed “double-seronegative”, is MS (16). False-negative results can be observed depending on the used methodology (e.g., live CBA vs. others) and the timing of when the sample was obtained for serological analysis (e.g., before or after PLEX, corticosteroids, or receiving immunotherapy) (2, 15). In this context, many neurologists have correctly identified the best assay for AQP4-IgG (i.e., live CBA) and the optimal timing to obtain the serum sample (e.g., during an attack). However, a study in LATAM has reported that the AQP4-IgG test was accessible only in 54% of countries and the MOG-IgG test was accessible only in 42% of countries (11). Such limitation in access to testing for AQP4-IgG and MOG-IgG poses challenges to the diagnosis of NMOSD in clinical settings (17). A recent study evaluated the frequency and factors associated with misdiagnosis of NMOSD in a cohort of patients from LATAM (13). The most frequent alternative diagnoses were MS (66.1%), clinically isolated syndrome (17.9%), and cerebrovascular disease (3.6%). NMOSD misdiagnosis was determined by MS/NMOSD specialists in 33.9% of cases. In addition, 86% of misdiagnosed patients were found to have an atypical MS syndrome; 50% of misdiagnosed patients had red flags (13). A study conducted in Poland, involving 1,112 patients with a suspected or confirmed diagnosis of acute or subacute onset of neurological deficits, examined the factors influencing the underdiagnosis of NMOSD (18); 18 patients had an established diagnosis of NMOSD, but 15 patients (83%) were initially misdiagnosed. The most common misdiagnosis was associated with idiotypic, monophasic, or non-specific demyelinating disease and MS. Atypical presentation, prolonged time to symptom development, overlap with other conditions, especially MS, and incorrect application or interpretation of diagnostic criteria were the main factors contributing to these findings. Additionally, variables such as gender, age of onset, and age of diagnosis may also play a significant role in the misdiagnosis process. In another study involving 199 NMOSD patients from the US, 71 were initially misdiagnosed (19). Factors associated with misdiagnosis included prolonged APS without other neurological symptoms, longer time to see a specialist, and delays in MRI acquisition. A greater proportion of misdiagnosed patients were identified with a negative live-CBA AQP4-IgG serum test result, 13/13 (100%) compared with 22/114 (19.3%). The time between first negative and subsequent positive AQP4-IgG tests was longer for misdiagnosed patients (19). These results are in line with surveys of MS specialists and non-specialist neurologists in the UK (20) and the US (21, 22) regarding the application of McDonald diagnostic criteria for MS that revealed numerous challenges related to understanding typical MS syndromes and applying “objective evidence” of a CNS MS-typical lesion, along with a low level of implementation in clinical practice.

Recently, a systematic review was conducted to identify reports of patients with non-demyelinating disorders that mimicked or were misdiagnosed with NMOSD. A total of 68 patients were included and 56 (82%) patients did not fulfill the 2015 NMOSD diagnostic criteria (7). The clinical syndromes misinterpreted for NMOSD were myelopathy (41%), myelopathy + optic neuropathy (41%), optic neuropathy (6%), or other (12%). Alternative etiologies included genetic/metabolic disorders, neoplasms, infections, vascular disorders, spondylosis, and other immune-mediated disorders. Common red flags associated with misdiagnosis were lack of cerebrospinal fluid (CSF) pleocytosis (57%), lack of response to immunotherapy (55%), progressive disease course (54%), and lack of MRI gadolinium enhancement (31%) (7). The most common conditions leading to misdiagnosis such as MS lack specific diagnostic biomarkers. The ultimate diagnosis relies on accurately recognizing and interpreting clinical symptoms, MRI results, and clinical expertise. As previously mentioned, misdiagnosis of NMOSD has not only been observed in LATAM; international data have highlighted that missed diagnosis or misdiagnosis of NMOSD also occur in other regions of the world.

The N-MOmentum trial (23) included both AQP4-IgG-seropositive and -seronegative patients to encompass a wide range of patient phenotypes diagnosed with NMO or NMOSD. AQP4-IgG-seronegative participants were required to meet the NMO clinical threshold according to the 2006 criteria. The independent eligibility committee (EC) assessed 50 AQP4-IgG-seronegative cases and determined that only 18 (36.0%) met the 2006 criteria; 10 patients were unanimously confirmed by members of the committee. Nearly two-thirds of potential AQP4 participants reviewed by the EC were deemed not eligible for randomization, despite all participants having an existing diagnosis of NMOSD at screening. Key reasons for exclusion included inadequate history of ON or myelitis, unclear MRI images, absence of LETM evidence, and inaccuracies in AQP4-IgG tests. Alternative diagnoses such as MS or sarcoidosis also led to exclusion. The primary reason for exclusion was the lack of LETM on MRI in 75% of cases (24 out of 50 AQP4-IgG-seronegative participants). Seven of 18 participants meeting the 2006 criteria and included in the clinical trial were later diagnosed with MOGAD (24); the 2015 NMOSD diagnostic criteria were developed before MOGAD was established as a disease distinct from AQP4-IgG-associated NMOSD. These observations underscore diagnostic challenges in seronegative NMOSD even when considered by an expert selection committee.

In the present study, we considered lack of awareness and education about the disease as a primary explanation for misunderstanding and misapplication of the diagnostic criteria for NMOSD. Approximately 60% of responses in this study were those of neurologists with subspeciality training in neuroimmunology. NMOSD is a rare disease, and repeated training and experience are required to appropriately identify clinical symptoms, imaging, and laboratory results to adequately diagnose this condition. A potential area for improvement in this respect is collaboration between neuroradiologists and neurologists for interpretation of imaging results in the differential diagnosis of NMOSD. Another explanation is overlap with other diseases that results in mistakes in the diagnosis of the disease. NMOSD can present symptoms and radiologic findings that overlap with other conditions such as MS and other neuroinflammatory disorders, thus explaining part of the findings (8). An international study aimed to evaluate if expert clinicians approach the diagnosis and treatment of overlapping NMO/MS patients similarly. Twelve carefully chosen AQP4-IgG-negative patients represented various clinical presentations seen in an NMO clinic. A total of 27 experts in NMO and MS scrutinized detailed clinical vignettes, along with pertinent imaging and lab results. Diagnoses fell into four groups (NMO, MS, indeterminate, and other). Overall clinician agreement on diagnosis was moderate (po = 0.51), with individual patient consensus varying from 0.25 to 0.73. For nine cases, opinions swayed between NMOSD and MS diagnoses, while others were labeled as monophasic LETM, acute disseminated encephalomyelitis (ADEM), or recurrent isolated ON (RION). Key NMO features, like LETM, held more sway in diagnosis than those akin to MS, such as STM (25). Additionally, a limitation in the access to diagnostic tests and procedures in some areas of LATAM may explain the frequency of misdiagnosis as well (26). Considering the previous findings, a need for awareness and continuous education in NMOSD among healthcare professionals in LATAM is crucial for improving diagnoses. This could involve training programs, public awareness campaigns, and international collaborations to share knowledge and resources (27, 28).

It is important to note the limitations of this study that may have impacted the validity and generalizability of the research, including its cross-sectional design, which precluded evaluation of changes over time; causality could not be evaluated. Nonetheless, we utilized methods similar to those previously employed in many studies (20–22). Sampling bias, stemming from the recruitment of participants from the LACTRIMS database and specific working groups from different neurological associations, may have skewed the study’s findings by favoring individuals already immersed in NMOSD research. This approach may not fully represent the broader population of neurologists across LATAM, potentially underestimating the true understanding and application of NMOSD diagnostic criteria among practitioners who are less actively engaged in research initiatives. We recognize that interpretation of imaging results can be challenging and is often shaped by subjective experience, particularly among non-radiologists. Thus, in the absence of objective assessment such as artificial intelligence methods, correctness of imaging interpretation is inherently subjective. Additionally, self-selection bias, inherent in the voluntary nature of participation, may have influenced the study’s results by potentially attracting neurologists with a higher level of experience or interest in NMOSD. Such individuals may have been more inclined to participate due to their familiarity with the topic or their desire to contribute to research efforts in the field. Taking these limitations into account provides a comprehensive perspective on the potential constraints and implications of the study findings. Despite these limitations, we consider that this is a valuable first study examining comprehension and application of the 2015 NMOSD criteria core elements by neurologists in LATAM.

In conclusion, our research found discrepancies among specialists in LATAM regarding the interpretation of clinical and radiological features related to the 2015 NMOSD criteria. Along with refining diagnostic criteria and their interpretation, there is a need to implement targeted continuing medical education campaigns and training initiatives focused specifically on NMOSD for healthcare professionals, including physicians, nurses, and allied health professionals. Additionally, it is important to consider improving education for other frontline clinicians most likely to encounter patients presenting with NMOSD, including emergency room or urgent care, ophthalmology, optometry, gastroenterology, or family practice healthcare providers. A multidisciplinary approach that facilitates collaboration across various specialties, including neurology, ophthalmology, immunology, and radiology, is important. Our study underscores the challenges LATAM specialists face in accessing training programs. Improving educational opportunities for reducing diagnostic errors and enhancing patient care standards is also important not only in LATAM but also worldwide.

## Data Availability

The raw data supporting the conclusions of this article will be made available by the authors, without undue reservation.
