# Supplementary material for: Application and interpretation of core elements of the 2015 NMOSD diagnostic criteria in routine clinical practice
Source: Front Immunol. 2024 Dec 13;15:1515481. doi: 10.3389/fimmu.2024.1515481 (PMC11671362; doi:10.3389/fimmu.2024.1515481)
Supplement: Supplementary file 1 [file Table1.docx]

**Supplementary Table 1: LATAM countries**

|  | **N=106** |
| --- | --- |
| **Countries, n (%)**  Argentina  Uruguay  Paraguay  Brazil  Chile  Peru  Ecuador  Colombia  Venezuela  Panama  Honduras  Guatemala  Costa Rica  Mexico | 30 (28.3)  7 (6.6)  2 (1.8)  21 (19.2)  2 (1.8)  4 (3.7)  9 (8.4)  13 (12.2)  5 (4.7)  3 (2.8)  2 (1.8)  1 (0.9)  1 (0.9)  6 (5.6) |

**Table S2. Access to educational meeting or training**

|  |  |
| --- | --- |
| **Have you performed any subspecialty fellowship or other training or master's degree and/or a practice focus within neuroimmunology?, n (%)**  Yes  **If yes, have you performed it in LATAM region?, n (%)**  Yes  **LATAM countries, n (%)**  Argentina  Brazil  Colombia  Mexico | 64 (60.3)  30 (46.9)  15 (50)  10 (33.3)  3 (10)  2 (6.3) |
| **Access to international (outside from LATAM) educational meeting or training?, n (%)**  Yes | 83 (78.3) |
| **Agreement rate with the following statement: from 1 (Strongly disagree) to 10 (Strongly Agree)** | |
| **I would like to access to international (outside from LATAM) educational meeting or training, but it is impossible to cover the international cost to me, n (%)**  Mean, ±SD  1-3  4-6  7-10 | 8.1 ±2  7 (6.6)  16 (15.1)  83 (78.3) |
| **There are no grants or financial support from any funding agency in the public, commercial or non-profit sectors in LATAM to attend to international (outside from LATAM) educational meeting or training, n (%)**  Mean, ±SD  1-3  4-6  7-10 | 7.7 ±2.6  3 (2.8)  28 (26.4)  75 (70.8) |
| **I find the 2015 NMOSD criteria easy to understand and apply for the diagnosis of NMOSD in clinical practice, n (%)**  Mean, ±SD  1-3  4-6  7-10 | 8 ±1.6  0  9 (8.5)  97 (91.5) |
| **Reviewed the 2015 IPND NMOSD Criteria carefully since its publication in July 2015, n (%)**  Yes  No | 93 (87.7)  13 (12.3) |
| **Reviewed the manuscript for the 2015 IPND NMOSD Criteria carefully more than 2 times at least 1 month apart, n (%)**  Yes  No | 72 (67.9)  34 (32.1) |
| **Published a scientific article on NMOSD, n (%)**  Yes, one or more original papers  Yes, one or more case reports/case series  No | 32 (30.2)  17 (16.1)  57 (53.7) |
| **How many papers per year do you publish related to NMOSD (if yes), n (%)**  1-5  6-10 | 46 (93.9) 2 (6.1) |

**Supplementary Table 3: Clinical practice aspects of treating neurologists.**

| **In your clinical practice, before making a new diagnosis of NMOSD, how often do you perform/request:** | |
| --- | --- |
| **Serum evaluation**  Always  Often  Occasionally  Rarely  Never  Not reported | 88 (83.1)  11 (10.3)  1 (0.8)  1 (0.8)  1 (0.8)  4 (3.7) |
| **CSF evaluation**  Always  Often  Occasionally  Rarely  Never  Not reported | 54 (50.9)  2 (1.8)  18 (16.9)  22 (20.7)  5 (4.7)  6 (5.6) |
| **Orbital MRI**  Always  Often  Occasionally  Rarely  Never  Not reported | 51 (48.1)  12 (11.3)  26 (24.5)  5 (4.7)  1 (0.8)  11 (10.3) |
| **Cervical spinal cord MRI**  Always  Often  Occasionally  Rarely  Never  Not reported | 91 (85.8)  8 (7.6)  2 (1.8)  1 (0.8)  0  4 (3.7) |
| **Thoracic spinal cord MRI**  Always  Often  Occasionally  Rarely  Never  Not reported | 79 (74.5)  7 (6.6)  14 (13.2)  1 (0.8)  0  5 (4.7) |

**Survey***

***Correct responses are shown in bolt**

**As part of your clinical practice, do you sometimes diagnose patients with NMOSD?**

**Yes (include for participation)**

No (exclude from participation)

**Do you treat adults with NMOSD?**

**Yes (include for participation)**

No (exclude from participation)

**What is your age? ______**

**What is your gender?**

F

M

Other (please specify)

**In which city do you practice? ______**

**In which country do you practice? ______**

**Estimate the number of patients you diagnose with NMOSD per year _______**

**Approximately what percentage of your professional time involves the clinical care of patients?**

25% or less

26-50%

51-75%

Greater than 75%

**Approximately what percentage of the patients for which you provide ongoing care have NMOSD or CNS inflammatory disease other than MS?**

1-5%

5-15%

15-30%

Greater than 30%

**Approximately what percentage of the patients for which you provide ongoing care have MS?**

25% or less

26-50%

51-75%

Greater than 75%

**Do you work as a clinical neurologist primarily in (check all that apply):**

Academic medical center

Individual private practice

Group private practice

Other (please specify)

**What year did you graduate from neurology residency? ______**

**For how many years following completion of your training have you been involved in NMOSD health care?.......**

**Have you performed any subspecialty fellowship or other training or master's degree and/or a practice focus within neuroimmunology?**

Yes

No

**If yes, have you performed any subspecialty fellowship or other training or master's degree and/or a practice focus within neuroimmunology in LATAM region?**

Yes

No

**If yes, where?** List from Argentina to Mexico

**List up to 3 centers (and country) that you consider for tertiary referral of complex patients with NMOSD**

**…………………………………………………..**

**Do you have any access to international (outside from LATAM) educational meeting or training?**

Yes

No

**Please rate your agreement with the following statement:** I would like to access to international (outside from LATAM) educational meeting or training, but it is impossible to cover the international cost to me.

(Strongly disagree) 1……………….10 (Strongly Agree)

**Please rate your agreement with the following statement:** there are no grants or financial support from any funding agency in the public, commercial or non-profit sectors in LATAM to attend to international (outside from LATAM) educational meeting or training

(Strongly disagree) 1……………….10 (Strongly Agree)

**Please rate your agreement with the following statement:** “I find the 2015 NMOSD criteria easy to understand and apply for the diagnosis of NMOSD in clinical practice”

(Strongly disagree) 1……………….10 (Strongly Agree)

**Have you had a chance to carefully read the 2015 IPND NMOSD Criteria since it was published in July 2015?**

Yes

No

**Have you read the manuscript for the 2015 IPND NMOSD Criteria more than 2 times at least 1 month apart?**

Yes

No

**Have you personally published a scientific article on NMOSD?**

Yes, one or more original papers

Yes, one or more case reports/case series

Yes, one or more reviews

No

**If yes, how many papers per year do you publish** **related to NMOSD?**

1-5

6-10

11-25

26-50

51-100

**CLINICAL ASPECTS**

**CASE 1**

**39-year-old woman with 1 prior optic neuritis in the right eye (confirmed by an ophthalmologist) and 1 prior myelitis event with moderate residual weakness of the right leg.**

**Currently, she consults for severe weakness in both legs associated with sphincter disorders that was confirmed by the neurologist during examination. No other sign and symptoms.**

**Brain and spinal cord MRI**

**Brain MRI does not reveal any new T2 or enlarging gadolinium enhancing lesions. Cervical and thoracic spinal cord MRI show a new thoracic T2 lesion extending from T1 to T8 with gadolinium enhancement from T3 to T5.**

These findings are commonly seen in MS patients

**These findings are commonly seen in patients with NMOSD seropositive for AQP4-Ab**

These clinical manifestations are commonly seen in patients with NMOSD, but not the MRI findings

These clinical manifestations are not commonly seen in patients with NMOSD, but spinal MRI lesion is typically seen in NMOSD.

**Does this patient's presentation fulfill dissemination in space criteria for a diagnosis of NMOSD?**

**This patient’s presentation currently fulfills dissemination in space criteria**

This patient’s presentation currently **does not fulfill** dissemination in time criteria

**If a history of optic neuritis would not have been present, but visual evoked potential of the right eye was prolonged despite absence of clinical symptoms in that eye, would criteria for NMOSD diagnosis been satisfied?**

Yes

**No**

**Based on the data above, does this patient meet diagnostic criteria for NMOSD if seronegative for AQP4-IgG?**

No, this patient does not have a core clinical characteristic of NMOSD.

No, although this patient presents with a core clinical characteristic for NMOSD, the patient is AQP4-IgG seronegative.

**Yes, this patient presents with a core clinical characteristic for NMOSD, and history of other** **core clinical characteristics and has the necessary supportive MRI finding of an acute longitudinally extensive spinal cord lesion thus meeting dissemination in space criteria assuming that no better explanation exists even with AQP4-Ab negative/unknown**

No, this patient presents with a core clinical characteristic for NMOSD, but dissemination in space and dissemination in time criteria are not fulfilled, and therefore, the patient has not NMOSD.

**CASE 2**

**33-year-old woman with 1 prior optic neuritis in the right eye with poor recovery (confirmed by an ophthalmologist).**

**Currently, she consults for moderate weakness in both legs (asymmetric with left predominance) associated with sphincter disorders that was confirmed by the neurologist during examination. No other sign and symptoms.**

**Brain and spinal cord MRI**

**Brain MRI shows a short canalicular lesion in the right ON in T2 without gadolinium enhancing and brain was normal. Cervical and thoracic spinal cord MRI show a thoracic T2 lesion from T3 to T4 (central in the axial plane) with gadolinium enhancement over T3.**

**Should the patient be tested for AQP4-IgG?**

No, this patient does not have a core clinical characteristic of NMOSD.

**Yes, this patient had a history of a core clinical characteristic of NMOSD and she now has clinically and radiologically a short-transverse myelitis, which does not rule out NMOSD.**

This patient has a short-transverse myelitis on spinal MRI, which rules out NMOSD

No, this patient had a history of ON typical of MS and she now has clinically and radiologically a short-transverse myelitis, which rules out NMOSD.

**CASE 3**

**26-year-old woman with no relevant medical history of prior diseases. She developed intractable episodic nausea and vomiting of 3 days of evolution. She was evaluated by a gastroenterologist, but not improvement after symptomatic therapy was observed. The patient was treated with IV metilprednisolone with complete recovery.**

**Brain and spinal cord MRI**

**Brain MRI shows bilateral lesion involving the dorsal medulla. Cervical and thoracic spinal cord MRI was normal.**

**Should the patient be tested for AQP4-IgG?**

**Yes, this patient has a core clinical characteristic of NMOSD associated with area postrema lesions on MRI.**

No, this patient does not have a core clinical characteristic of NMOSD and false positives may be obtained in this setting

Yes, although this patient does not fulfill criteria for an area postrema syndrome.

No, this patient has a typical syndrome of MS.

**Although considered a core clinical characteristic for NMOSD, which manifestation is not a cardinal syndrome and would be insufficient to support a diagnosis of NMOSD in an AQP4-IgG seronegative patient for NMOSD? (Please select all that apply)**

- Optic neuritis
- Transverse myelitis
- **Brainstem syndrome**
- Area postrema syndrome
- **Diencephalic syndrome**
- **Symptomatic Cerebral syndrome**

**Which of the following are considered typical clinical presentations of NMOSD or highly suggestive for NMOSD? (Please select all that apply)**

- **Acute unilateral optic neuritis with a poor visual recovery**
- **Complete transverse myelopathy with bilateral motor and sensory involvement**
- Double vision due to an internuclear ophthalmoplegia (in a young adult < 40 years old)
- Headache or meningism
- Facial sensory loss or trigeminal neuralgia (in a young adult < 40 years of age)
- Partial myelopathy
- Complete gaze palsy or fluctuating ophthalmoparesis
- Isolated fatigue or asthenia
- **Bilateral optic neuritis or unilateral optic neuritis with a poor visual recovery**
- Subacute cognitive decline
- **Intractable nausea, vomiting, or hiccoughs**
- Lhermitte's symptom
- Urge incontinence or erectile dysfunction
- **Hypersomnolence or narcolepsy-like syndrome**

Which of the following regions may be used **as additional MRI requirements for NMOSD without AQP4-Ab and NMOSD with unknown AQP4-Ab**? (Check all that apply)

- **Long optic nerve lesion(s)**
- **Extensive periependymal brain lesions**
- Periventricular
- **Lesions involving the hypothalamus, thalamus**
- **Large, confluent subcortical or deep white matter lesion(s)**
- Cortical lesions
- **Lesions involving the dorsal medulla**
- Infratentorial lesions (i.e middle cerebellar peduncles)
- Fluffy infratentorial lesions
- Subcortical
- **Long, diffuse, heterogeneous, or edematous corpus callosum lesions**
- Juxtacortical
- **Spinal cord (i.e. lesions extending over 3 or more complete vertebral segments)**
- **Periependymal surfaces of the fourth ventricle**
- periventricular lesions extending perpendicularly from ventricles into brain white matter
- **Long lesions in corticospinal tract pathway**


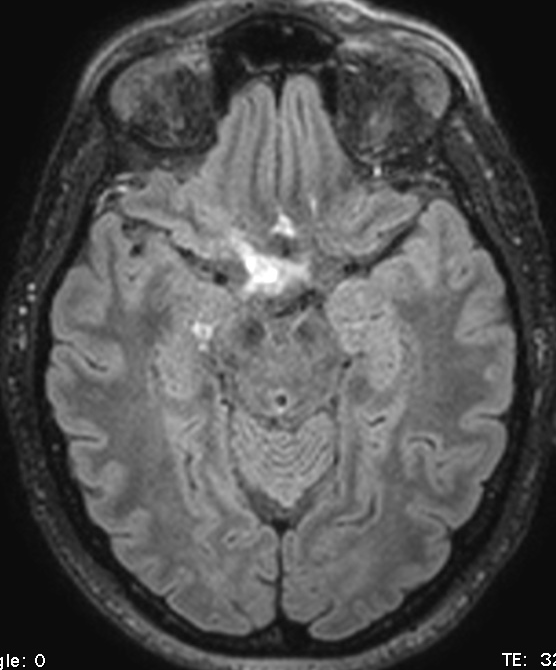

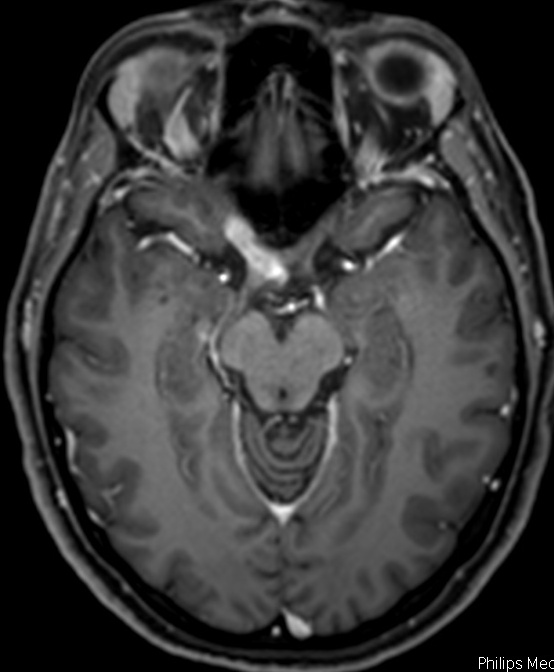


**Is this lesion considered as a typical lesion for NMOSD?**

**Yes**

No

What type of lesion is it?: List (this applies to all the following images):

- Longitudinal extensive optic nerve lesion(s)
- Extensive periependymal brain lesions
- Periventricular
- Lesions involving the hypothalamus
- Lesions involving the thalamus
- **Lesion involving optic chiasm and posterior ON**
- Large, confluent subcortical or deep white matter lesion(s)
- Cortical lesions
- Lesions involving the area postrema
- Infratentorial lesions (i.e middle cerebellar peduncles)
- Fluffy lesion and poorly demarcated lesion
- Subcortical
- Long, diffuse, heterogeneous, or edematous corpus callosum lesions
- Juxtacortical
- Temporal lobe lesion/s
- Spinal cord (i.e. lesions extending over 3 or more complete vertebral segments)
- Periependymal surfaces of the fourth ventricle
- periventricular lesions extending perpendicularly from ventricles into brain white matter
- Long lesions in corticospinal tract pathway


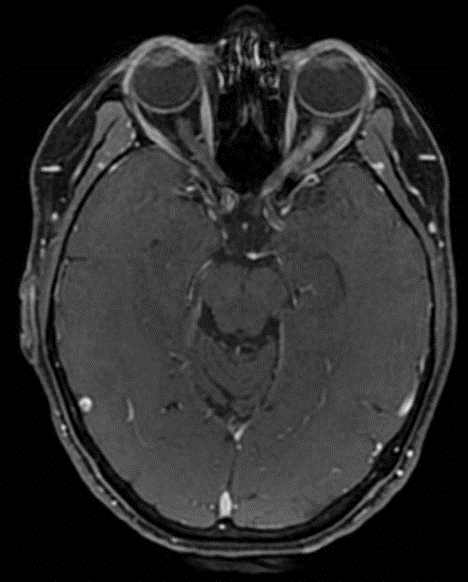


**Is this lesion considered as a typical lesion for NMOSD?**

**Yes**

No

What type of lesion is it?: List: **LEON (longitudinally extensive left optic neuritis)**


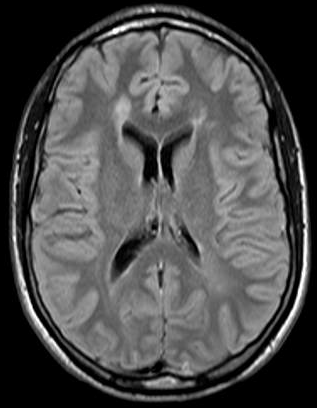


**Is this lesion considered as a typical lesion for NMOSD?**

Yes

**No**

What type of lesion is it?: List: **Periventricular (MS)**


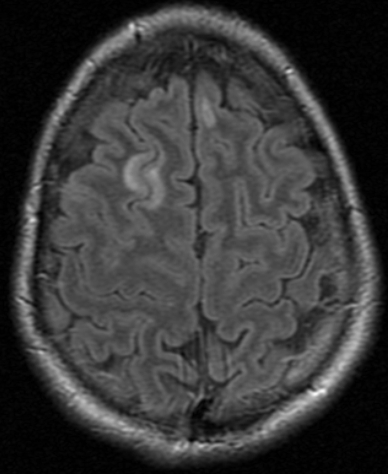


**Is this lesion considered as a typical lesion for NMOSD?**

Yes

**No**

What type of lesion is it?: List **Juxtacortical (MS)**


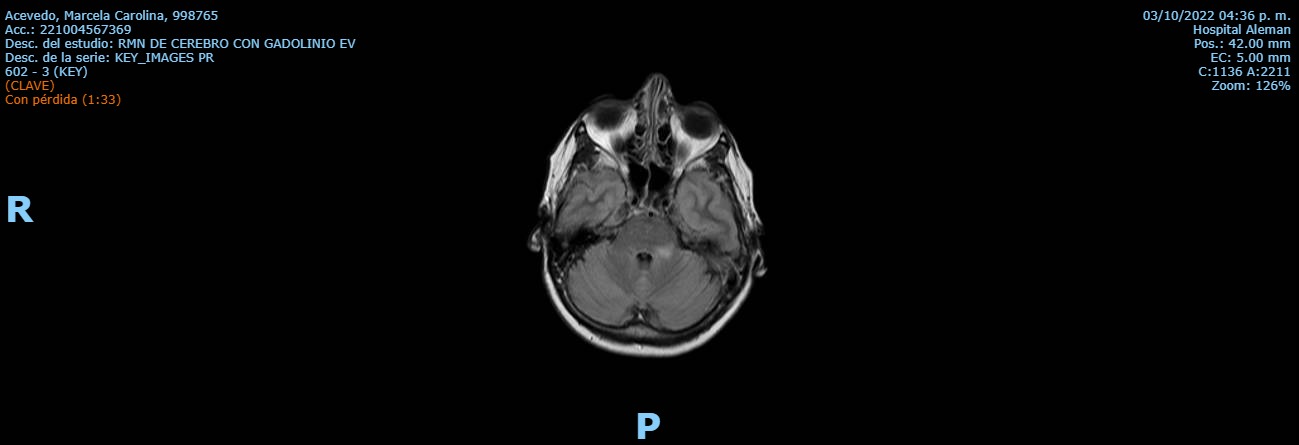


**Is this lesion considered as a typical lesion for NMOSD?**

Yes

**No**

What type of lesion is it?: List **Fluffy lesion and poorly demarcated lesion (MOGAD)**


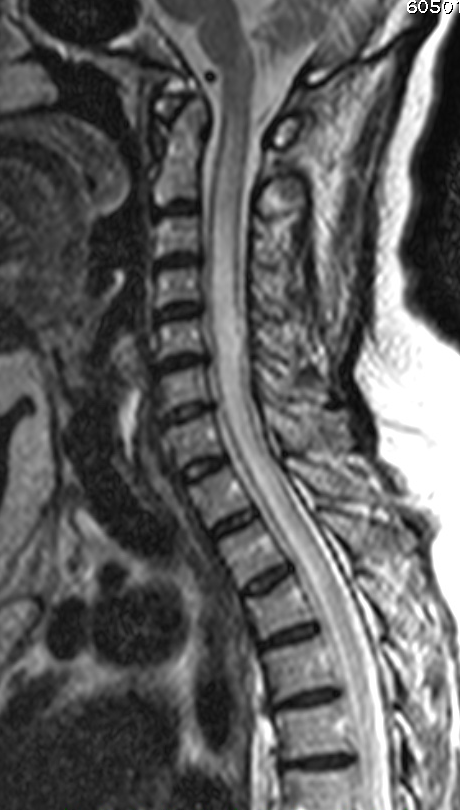


**Is this lesion considered as a typical lesion for NMOSD?**

**Yes**

No

What type of lesion is it?: List: **LETM**


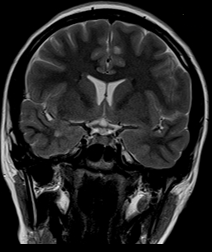


**Is this lesion considered as a typical lesion for NMOSD?**

Yes

**No**

What type of lesion is it?: List: **temporal lobe (MS)**


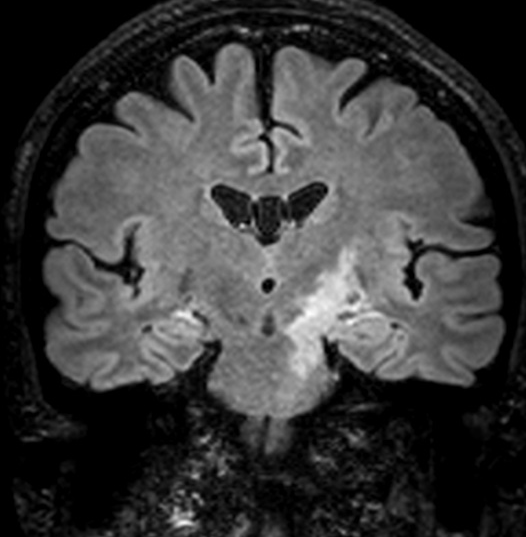


**Is this lesion considered as a typical lesion for NMOSD?**

**Yes**

No

What type of lesion is it?: List: **Corticospinal tract lesion**


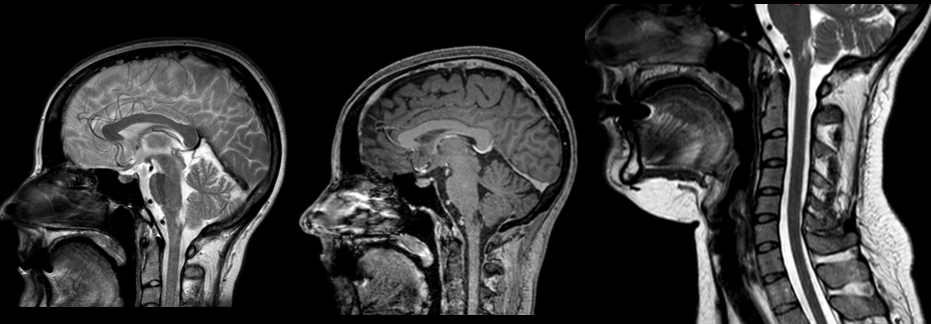


**Is this lesion considered as a typical lesion for NMOSD?**

**Yes**

No

What type of lesion is it?: List: **Lesion involving the area postrema**


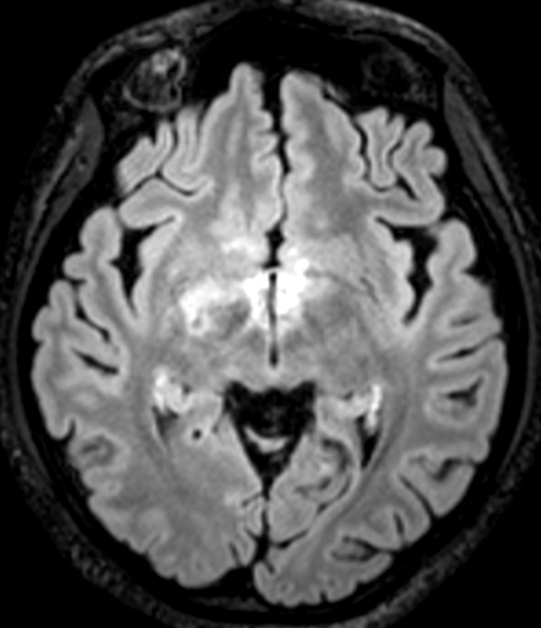


**Is this lesion considered as a typical lesion for NMOSD?**

**Yes**

No

What type of lesion is it?: List: **hypothalamic lesion**


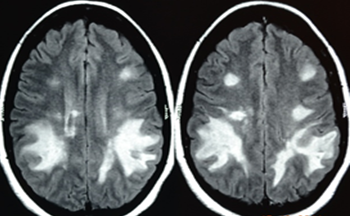


**Is this lesion considered as a typical lesion for NMOSD?**

**Yes**

No

What type of lesion is it?: list: **Large, confluent, bilateral subcortical or deep white matter lesions**

**To fulfill NMOSD diagnostic criteria, seronegative patients must experience 2 or more different core clinical characteristics (i.e., dissemination in space, affecting different neuroanatomic regions) and other supportive MRI characteristics must also be present**

False

**True**

**According the 2015 NMOSD criteria: Area postrema syndrome is classified as: Episode of 1) hiccups or 2) nausea and vomiting; occurring most of each day for at least 2 days, with or without additional neurological symptoms and not attributable to other cause; 12 hours duration suffices if associated with new/acute area postrema/dorsal medulla MRI lesion.**

**False**

True

**Acute myelitis with extension into the brainstem can establish DIS (two different neuroanatomic regions)**

**False**

True

**In clinical practice, diagnosis of NMOSD is not possible if OC bands are found with a pattern II or III (e.g. in a patient with severe ON and poor recovery + positivity for OCB).**

**False**

True

**Which of the following regions are considered as red flags (conventional neuroimaging) for NMOSD?** (Check all that apply)

- - Long optic nerve lesion(s)
  - Extensive periependymal brain lesions
  - **Periventricular**
  - Lesions involving the hypothalamus, thalamus
  - Large, confluent subcortical or deep white matter lesion(s)
  - **Cortical lesions**
  - **Lesions adjacent to lateral ventricle in the inferior temporal lobe**
  - Lesions involving the dorsal medulla
  - Infratentorial lesions (i.e middle cerebellar peduncles)
  - Fluffy infratentorial lesions
  - Subcortical
  - Long, diffuse, heterogeneous, or edematous corpus callosum lesions
  - **Juxtacortical**
  - **Spinal cord (i.e. peripheral lesion extending over <1 vertebral segment)**
  - Periependymal surfaces of the fourth ventricle
  - **Dawson fingers**
  - Corticospinal tract lesions
  - **Lesions with persistent (>3 months) gadolinium enhancement**

In your clinical practice, **before** making a new diagnosis of NMOSD, how often do you perform/request:

**Serum evaluation**

Always

Often

Occasionally

Rarely

Never

**Which of the following serologic tests would you perform if a phenotype of NMOSD is observed? (Check all that apply)**

- AQP4-Ab only in serum
- MOG-Ab only in serum
- **AQP4-Ab and MOG-Ab in serum (at the same time if possible)**
- AQP4-Ab only in CSF
- MOG-Ab only in CSF
- AQP4-Ab and MOG-Ab in serum and CSF (at the same time)
- AQP4-Ab and MOG-Ab in CSF (at the same time)

**What is the optimal method for testing AQP4-IgG to maximize sensitivity and specificity?**

- Tissue-based IFI
- **Live cell-based assay**
- ELISA
- Fixed cell-based assay
- Flow cytometry

**When (optimal moment) would you request AQP4-Ab during the NMOSD diagnosis process (before making a new diagnosis of NMOSD)? (Check all that apply) *responses will be randomly ordered per respondent**

- **During an attack**
- After receiving immunosuppressant treatments
- In remission phase
- **90 days after completion of treatment if PLEX or IVMP was received**
- **Before receiving immunosuppressant treatments**
- **After 3-6 months if initial AQP4-Ab were negative, but high suspicion of NMOSD exists.**
- Before 30 days if PLEX or IVMP was received

**CSF evaluation**

Always

Often

Occasionally

Rarely

Never

**Orbital MRI**

Always

Often

Occasionally

Rarely

Never

**Cervical spinal cord MRI**

Always

Often

Occasionally

Rarely

Never

**Thoracic spinal cord MRI**

Always

Often

Occasionally

Rarely

Never

**Acknowledgment**

We would kindly like to thank all colleagues who participated in the survey: Dr Jefferson Becker (PUCRS, Brazil); Dr Gustavo Figueira (Hospital São Francisco de Assis, Brazil); Dr Alex Eduardo da Silva (Universidade Federal do Triângulo Mineiro, Brazil); Dr Satiko Andrezza Ferreira Takano (Federal University of Amazonas, Brazil); Dr Claudia Cristina Ferreira Vasconcelos (Universidade Federal do Estado do Rio de Janeiro-UNIRIO, Brazil); Dr Giordani Rodrigues dos Passos (Pontifical Catholic University of Rio Grande do Sul, Brazil); Dr Ana Clara Guilherme Rodrigues (Centro de Referência em Esclerose Múltipla da Paraíba, Brazil); Dr Marianna Moraes (UNIFESP, Brazil); Dr Anderson Kuntz Grzesiuk (Clinica Nossa Senhora das Graças, Cuiabá, Brasil); Dr Juliana Mathias Netto Khouri (Matheus Wasem, Brazil); Dr Fernando Tenório Gameleira (University Hospital Federal University of Alagoas, Brazil); Dr Daissy Liliana Mora Cuervo (Hospital Moinhos de Vento, Brazil); Dr Fernando Figueira (Hospital São Francisco na Providencia de Deus

Thiago Santos Nascimento (Hospital Universitário de Sergipe, Mestrando em Ciências da Saúde pela UFBA, Brazil); Dr Anderson Kuntz Grzesiuk (Private clinic, Brazil); Dr Fernando Gracia (Hospital Santo Tomas, Panamá); Dr Valeria Rocha (Hospital Policial and Hospital Británico, Uruguay); Dr Sindy Segovia (Hospital Central Ips, Paraguay); Dr Graciana L Galiana (Hospital Lagomaggiore, Argentina); Dr Eduardo Duriez (Sotelo doctors Hospital Monterrey, Mexico); Dr Eunice Ramírez (Hosp Dr. Mario Rivas, Honduras); Dr Silvio Chaves Huertas (Fundación Hospital San Pedro Pasto, Colombia); Dr Jorge Luis Wong Armas (Hospital Maria Auxiladora, Peru); Dr Mauro Stagno (Comepa, Uruguay); Dr Alejandro Díaz (Instituto Guatemalteco de Seguridad Social, Guatemala); Dr Mariana de Virgiliis (Hospital Universitario Austral/ Hospital Oftalmológico Lagleyze, Argentina); Dr Diana Maria Parada Palacios (NEUROCAD IBAGUE, Colombia); Dr Eduardo Galli (CENTRO MEDICO AMUR, Paraná); Dr Macarena Clementi (Hospital de San Isidro, Argentina); Dr Roberto Rotta Escalante (Policlinica Bancaria, Argentina); Dr Cesar A Franco Ruiz (Fundación Instituto Neurológico de Colombia); Dr Paula Goyeneche (Hospital del Carmen, Argentina); Dr Johnny Andres Lopez Garcia (Universidad de Medellin, Colombia); Dr Juan Pablo Mansilla (Universidad de Valparaíso, Chile); Dr Victor Paredes (Fundación Ecuatoriana de Esclerosis Múltiple); Dr José Ignacio Gortari (Medicarte, Colombia); Dr Gabriela Acuña Chong (Hospital Teodoro Maldonado Carbo, Ecuador); Dr Juan D. Farfán-Albarracín (HOMI Fundación Hospital Pediátrico La Misericordia, Colombia); Dr Ariadna Silva-Lepe (Oftari, Mexico); Dr Hyland Arroyo (Universidad Central Del Ecuador); Dr Nayeli Sánchez Rosales (Centro Médico Nacional de Occidente, Mexico); Dr Dolores Loor Alcivar (Hospital de Especialidades Portoviejo, Ecuador); Dr Antonio Schlesinger (Colombia); Dr Maria Isabel Reyes Mantilla (Hospital Simon Bolivar, Colombia); Dr Maria Eugenia Balbuena Aguirre (Hospital de Clinicas, Argentina); Dr Gloria Rosario Michelena Bustamante (Clínica 25 de Mayo, Argentina); Dr Jairo Quiñones (Fundación Valle del Lili, Colombia); Dr Nora Fernández Liguori (Sanatorio Güemes, Argentina); Dr Nicia Eunice Ramírez (Hosp Dr. Mario Rivas, Honduras); Dr Graciana Lourdes Galiana (Mendoza, Argentina); Dr Irene Treviño Frenk (UNAM, Mexico); Dr Ibis Soto de Castillo (Hospital Maracaibo, Venezuela); Dr Analisa Manin (Hospital de Agudos J. M. Ramos Mejía, Argentina); Dr. Eli Skromne (Mexico); Dr Lorna Galleguillos (Clínica Alemana, Chile); Dr Guido Rinaldi (HIGA San Martin de La Plata, Argentina); Dr Arón Benzadón (Ciudad de la Salud, Panamá); Dr Vladimiro Sinay (Fundación Favaloro / INECO, Argentina); Dr Cecilia Adriana Gonzalez (Hospital Ramos Mejía, Argentina); Dr Sheila Castro-Suarez (Instituto Nacional de Ciencias Neurológicas, Perú); Dr Flabia Ramirez (Caracas, Venezuela); Dr Maria Agustina Piedrabuena (FLENI, Argentina); Dr Peñalver Francisco Javier (Hospital Lagomaggiore, Argentina); Dr Amelia Alves Pinheiro (Hospital San Martin de Paraná, Argentina); Dr Edgar Rojas (Hospital Guillermo Almenara essalud, Peru); Dr Felipe Castro (Clinica La Estancia, Colombia); Dr Myrian Zudaire (HIGA DR Oscar E. Allende, Argentina); Dr Geraldine Luetic (Instituto de Neurociencias de Rosario, Argentina); Dr Carolina Mainella, Hospital Español de Rosario, Argentina)

Dr. Luis Enrique Molina Carrión (, Centro Médico Nacional La Raza, México)
